# Anterior Cingulate Cortex Glutamate Levels Are Related to Response to Initial Antipsychotic Treatment in Drug-Naive First-Episode Schizophrenia Patients

**DOI:** 10.3389/fpsyt.2020.553269

**Published:** 2020-10-23

**Authors:** Jinguang Li, Honghong Ren, Ying He, ZongChang Li, Xiaoqian Ma, Liu Yuan, Lijun Ouyang, Jun Zhou, Dong Wang, Chunwang Li, Xiaogang Chen, Hongying Han, Jinsong Tang

**Affiliations:** ^1^Hunan Key Laboratory of Psychiatry and Mental Health, Department of Psychiatry, National Clinical Research Center for Mental Disorders, Hunan Medical Center for Mental Health, China National Technology Institute on Mental Disorders, Institute of Mental Health, The Second Xiangya Hospital, Central South University, Changsha, China; ^2^Affiliated Wuhan Mental Health Center, Tongji Medical College of Huazhong University of Science and Technology, Wuhan, China; ^3^Department of Psychiatry, Suzhou Psychiatric Hospital, The Affiliated Guangji Hospital of Soochow University, Suzhou, China; ^4^Department of Radiology, Hunan Childen's Hospital, Changsha, China; ^5^Department of Psychiatry, The Third Affiliated Hospital of Sun Yat-Sen University, Guangzhou, China; ^6^Department of Psychiatry, Sir Run-Run Shaw Hospital, School of Medicine, Zhejiang University, Hangzhou, China

**Keywords:** glutamate, magnetic resonance spectroscopy, ACC, anterior cingulate cortex, Cr, creatine, FES, first-episode schizophrenia

## Abstract

The glutamatergic system has previously been shown to be involved in the pathophysiology of schizophrenia and the mechanisms of action of antipsychotic treatment. The present study aimed to investigate the relationship between the levels of glutamate (Glu) or Glu/total creatine (Glu/Cr+PCr) in the anterior cingulate cortex (ACC) and psychiatric symptoms as well as the response to antipsychotic treatment. We performed proton magnetic resonance spectroscopy (^1^H–MRS) to measure Glu and Glu/Cr+PCr in the ACC of 35 drug-naïve first-episode schizophrenia (FES) patients and 40 well-matched healthy controls (HCs). After scanning, we treated the patients with risperidone for eight weeks. Remission status was based on the Positive and Negative Syndrome Scale (PANSS) scores at week 8. At baseline, there were no significant differences in the levels of Glu or Glu/Cr+PCr in the ACC between drug-naïve FES patients and HCs. Lower baseline levels of Glu/Cr+PCr but not Glu in the ACC were associated with more severe negative symptoms of schizophrenia. Compared to the remission group (RM), the non-remission group (NRM) had lower baseline ACC Glu levels (*P* < 0.05). Our results suggest that ACC Glu levels may be related to the severity of symptoms in the early stages of schizophrenia and therefore may be a marker with which to evaluate the treatment effect of antipsychotics in schizophrenia patients.

## Introduction

Schizophrenia is a complex and severe mental disorder with a lifetime prevalence of 1% worldwide (1). Although it has been over 60 years since the first antipsychotic, chlorpromazine, was initially developed, a third of patients with schizophrenia have a suboptimal response to first-line antipsychotic treatment, most of which mainly target dopamine D2 receptors (2, 3). The dopaminergic hypothesis can partially explain the psychopathology of positive symptoms in schizophrenia. However, dopaminergic antipsychotics have only a limited effect on negative symptoms and cognitive deficit, which are the best predictive factors of persistent disability and a poor response to antipsychotics (4, 5).

Converging lines of evidence from pharmacological and neuropharmacological studies suggest that glutamatergic dysfunction also contributes to deficits in schizophrenia (6–8). Studies have shown that administration of phencyclidine (PCP), dizocilpine (MK801), and ketamine, antagonists of the N-methyl-d-aspartate glutamate receptor (NMDAR), to healthy volunteers or rodents can induce schizophrenia-like symptoms (9–11). In pharmacological animal models of schizophrenia, compared with other drugs, such as amphetamine, NMDAR antagonists can cause positive and negative symptoms that more closely mimic those of schizophrenia (12). Genetic animal models also provide a wealth of data showing that decreased NMDAR activity can lead to changes in the brain and behavior, similar to those observed in schizophrenia (13). First-episode schizophrenia studies have shown that approximately one-quarter of first-episode schizophrenia (FES) patients have persistent psychosis symptoms despite adequate initial treatment (14), and this finding, together with the glutamatergic hypothesis, may suggest that dopaminergic drugs are ineffective throughout illness in the subgroup of schizophrenia patients, who have an inadequate response to initial treatment with dopaminergic drugs.

Proton magnetic resonance spectroscopy (^1^H-MRS) is an efficient and non-invasive method for detecting the concentration of Glu in the human brain. Many studies have found differences in glutamate levels in target areas of the brain between schizophrenia patients and healthy controls (15–18), and glutamate levels have been associated with the severity of symptoms and cognitive function (19–21). These studies have aroused great interest in whether the levels of glutamate in the brain can predict the response to antipsychotics and the outcome of schizophrenia. The current hypothesis is that inadequate response to conventional dopaminergic antipsychotics is more likely to be associated with the glutamatergic system (22). In previous ^1^H-MRS studies, the results of measurements of glutamate levels in the brains of schizophrenia patients were mixed (23), and the findings of the relationship between glutamate levels and the severity of symptoms of schizophrenia were also inconsistent (24). The discrepancy of the above results may be due to the effect of age, stage of the disease, and treatment with antipsychotics (25, 26).

In this study, we implemented a longitudinal design to explore the relationship between glutamate levels and symptom severity as well as the response to antipsychotics. To reduce the effect of the confounding factors mentioned above, we selected drug naïve first-episode psychiatric patients as the experimental cohort. In previous studies, the ACC has been shown to play a critical role in cognitive impairment and psychotic symptoms in schizophrenia patients (27, 28). Therefore, we selected the ACC as the target region. At baseline, ^1^H-MRS scans were performed on patients and healthy controls who met the study criteria. We assessed the symptom severity of the patients using the PANSS scale at both baseline and week 8 after risperidone treatment. We chose risperidone as the administered antipsychotic because it is widely prescribed and very inexpensive in China. We expected to determine whether baseline levels of Glu or Glu/Cr+PCr in the ACC are associated with the severity of psychiatric symptoms and with the response to initial antipsychotic administration in drug naïve FES.

## Results

### Comparison of Demographic Characteristics and Metabolite Levels Between HC and FES Patients

The demographic characteristics and metabolite levels are summarized in Table 1. Thirty-five FES patients met the inclusion criteria, were first diagnosed with schizophrenia, and had never been treated with antipsychotics. There was no significant difference in age, gender, education, or tobacco or alcohol use between drug-naïve FES patients and HCs. As shown in Table 1, no significant differences were observed in Glu and Glu/Cr+PCr levels in the ACC between HCs and FES patients at baseline. There were no significant differences in the Glu and Glu/Cr+PCr levels of the males and females between the patient group and the control group. Both *p*-values were higher than 0.05 (Supplementary Table 1).

**Table 1:** Demographic, clinical characteristics and MRS data of controls and schizophrenia patients.

	**Controls (*n* = 40)**	**Patients (*n* = 35)**	***P*-value**
Age, mean ± SD, y	22.75 ± 4.19	22.286 ± 4.46	0.643
Gender (male/female)	25/15	22/13	0.975
Education, mean ± SD, y	11.50 ± 1.68	11.06 ± 2.33	0.343
Handedness (right/left)	40/0	35/0	-
Tobacco use (yes/no)	6/29	10/30	0.407
Alcohol use (yes/no)	0/40	0/35	-
ACC-Glu, mean ± SD	8.79 ± 0.91	8.92 ± 1.14	0.572
ACC-Glu/Cr+PCr, mean ± SD	1.61 ± 0.18	1.65 ± 0.27	0.413
ACC-FWHM, mean ± SD	0.065 ± 0.021	0.069 ± 0.017	0.339
ACC-S/N, mean ± SD	24.73 ± 2.91	23.97 ± 2.70	0.251

*ACC, anterior cingulate cortex; Glu, Glutamate; Cr, creatine; PCr, phosphocreatine; FWHM, full width at half maximum; S/N, signal-noise-noise ratio*.

### Quality of ^1^H-MRS Spectra

The mean ± SD of the FWHM from the LC Model in the ACC was 0.069 ± 0.017 ppm for the FES patients and 0.065 ± 0.021 ppm for the HCs. The mean ± SD of the S/N in the ACC was 23.97 ± 2.70 for FES patients and 24.73 ± 2.91 for HCs. There were no significant differences in the FWHM and S/N in the target voxels between FES patients and HCs (FWHM, T73 = 0.849 *p* = 0.399; S/N, T73 = −1.156, *p* = 0.251). The CRLB% values of Glu, Cr, and PCr in the ACC for both the FES patients and HCs were <20%. Data on the quality of the ^1^H-MRS spectra are summarized in Table 1.

### Correlations of Metabolite Levels With Clinical Symptoms

**Figure 1:**
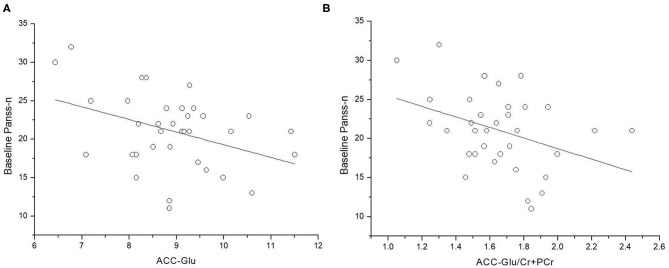
Relationship between levels of glutamate (Glu and Glu/Cr+PCr) in anterior cingulate cortex (ACC) and PANSS negative symptom scores. **(A)** ACC-Glu and PANSS negative symptom scores at baseline (*n* = 35, *r* = −0.360, *p* = 0.034). **(B)** ACC-Glu/Cr+PCr and PANSS negative symptom scores at baseline (*n* = 35, *r* = −0.432, *p* = 0.010).

At baseline, lower levels of Glu and Glu/Cr+PCr in ACC were associated with more severe negative symptoms (Figure 1) (Glu and PANSS-n: *r* = 0.360, *p* = 0.034; Glu/Cr+PCr and PANSS-n: *r* = −0.432, *p* = 0.010). After controlling for age, the relationship between the Glu level in the ACC and negative symptoms was not significant (*r* = −0.323, *p* = 0.062), but lower Glu/Cr+PCr levels remained associated with a higher PANSS-n score (*r* = −0.446 *p* = 0.008).

### Remission vs. Non-remission Patients

According to the standards proposed by the Remission in Schizophrenia Working Group (29), 25 patients (71%) met the remission criteria, and 10 patients (29%) were in non-remission status. Non-remission patients were younger, had more severe symptoms, and were treated with a higher dose of risperidone during the study (Table 2).

**Table 2:** Demographic, clinical characteristics, and MRS data of remission and non-remission.

	**Remission (25)**	**Non-remission (10)**	***P*-value**
Age, mean ± SD, y	23.48 ± 4.52	19.30 ± 2.58	0.010
Gender (male/female)	14/11	8/2	0.259
Risperidone dose, mean ± SD	3.60 ± 0.50	4.30 ± 0.48	0.001
Education, mean ± SD, y	11.24 ± 2.49	10.60 ± 1.90	0.470
(Baseline)Panss-T	80.68 ± 12.11	94.50 ± 13.55	0.006
(Baseline)Panss-P	21.88 ± 4.71	24.60 ± 6.20	0.168
(Baseline)Panss-N	16.68 ± 3.52	22.60 ± 4.17	<0.001
(Baseline)Panss-G	42.12 ± 8.91	47.30 ± 7.71	0.117
(Week8) Panss-T	53.72 ± 8.27	81.40 ± 9.88	<0.001
(Week8) Panss-P	11.36 ± 2.40	20.40 ± 5.21	<0.001
(Week8) Panss-N	11.88 ± 2.35	19.60 ± 4.25	<0.001
(Week8) Panss-G	30.48 ± 1.16	41.40 ± 5.68	<0.001
ACC-Glu	9.22 ± 1.05	8.17 ± 1.08	0.013
ACC-Glu/Cr+PCr	1.67 ± 0.26	1.53 ± 0.28	0.117

*PANSS, Positive and Negative Symptoms Scale; PANSS-T, PANSS total scores; PANSS-P, PANSS positive symptom scores; PANSS-N, PANSS negative symptom scores; PANSS-G, PANSS general psychopathological symptom scores*.

**Figure 2:**
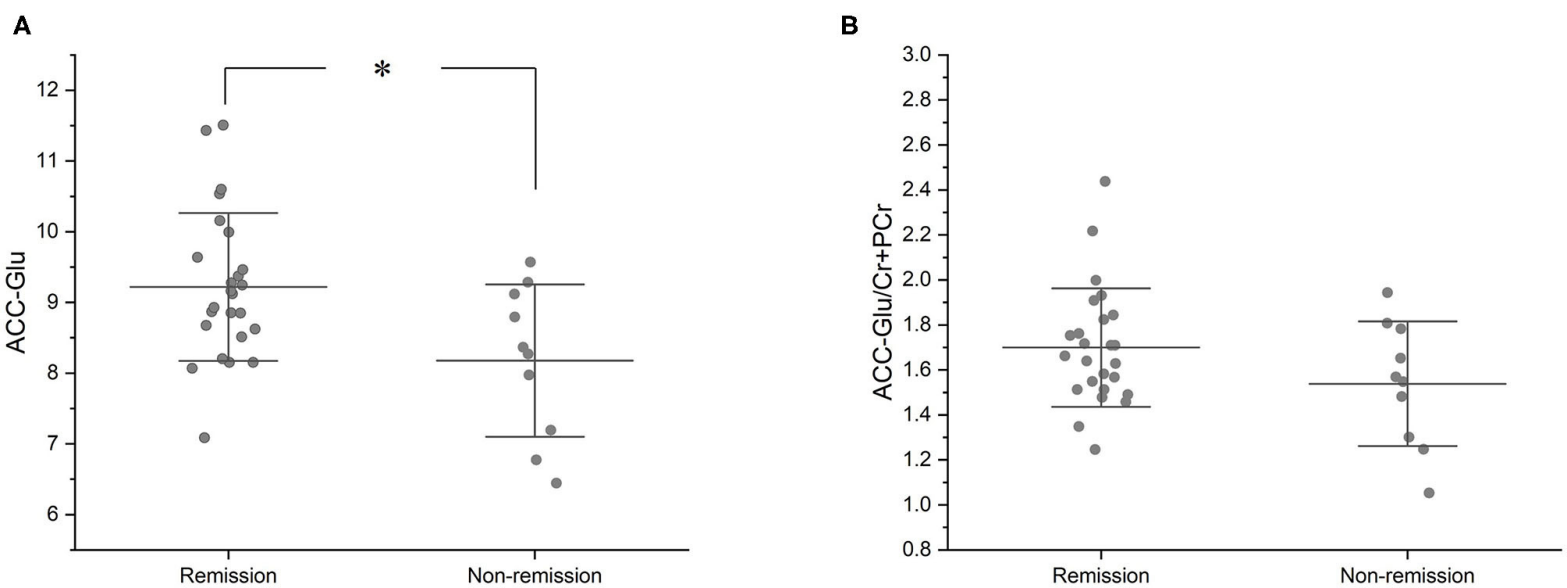
Baseline Glu and Glu/Cr+PCr in ACC of remission and non-remission groups. **(A)** At baseline, the remission group had a significantly higher Glu level than the non-remission group (mean values, 9.22 ± 1.05 vs. 8.17 ± 1.08; T33 = −2.634, *P* = 0.013). **(B)** At baseline, the remission group had a higher Glu/Cr+PCr than the non-remission group, but there was no statistically significant difference between the two groups (mean values, 1.67 ± 0.26 vs. 1.53 ± 0.28; T33 = −1.612, *p* = 0.117). *p < 0.05.

Compared with the remission group, non-remission patients had a significantly lower Glu level in the ACC (T33 = −2.634 *P* = 0.013). However, the difference in Glu/Cr+PCr levels in the ACC between the remission and non-remission groups was not significant (1.67 ± 0.26 vs. 1.53 ± 0.28, T33 = −1.612, *p* = 0.117) (Figure 2, Table 2).

### Prediction of Non-remission

To distinguish between remission and non-remission patients, we implemented a binary logistic regression model. Baseline Glu or Glu/Cr+PCr in the ACC was associated with the likelihood of remission in FES patients after treatment with risperidone. In the Glu model (Nagelkerke R^2^ = 0.256, B = −1.048, Wald = 4.945, *p* = 0.026, EXP[B] = 0.351), 77.1% of FES patients (96% remission patients and 30% non-remission patients) were correctly segregated. In the Glu/Cr+PCr model (Nagelkerke R^2^ = 0.110, B = −2.602, Wald = 2.367, *p* = 0.124, EXP[B] = 0.074), 74.3% of FES patients (96% remission patients and 20% non-remission patients) were correctly segregated. When age and PANSS-total score were included in the two models, the accuracy increased to 85.7% (92% remission patients and 70% non-remission patients, Nagelkerke R^2^ = 0.674) in the model with baseline Glu and 82.9% (88% remission patients and 70% non-remission patients, Nagelkerke R^2^ = 0.655) in the model with baseline Glu/Cr+PCr (Table 3).

**Table 3:** Models of binary logistic regression.

**Models**	**Nagelkerke R^**2**^**	**HL-test**	**Accuracy of Re (%)**	**Accuracy of non-Re (%)**	**Overall Accuracy (%)**
Model 1	0.256	0.895	96	30	77.1
Model 2	0.110	0.472	96	20	74.3
Model 3	0.670	0.987	92	70	85.7
Model 4	0.561	0.477	88	70	82.9

*HL-test, Hosmer–Lemeshow test; Re, remission; non-Re, non-Remission*.

*Model 1: Glu; Model 2: Glu/Cr+PCr; Model 3: Glu; Age; Panss-total score; Model 4: Glu/Cr+PCr; Age; Panss-total score*.

## Discussion

Glutamate is the most important excitatory neurotransmitter in the nervous system (30), and NMDAR is widely distributed throughout the human brain. The glutamate measurement provided by ^1^H-MRS reflects the amount of glutamate in the target region in the brain and is also considered an indicator of motor cortical excitability (31, 32). Previous studies have observed that phencyclidine (PCP) and ketamine, which are antagonists of NMDAR, can transiently induce positive psychotic symptoms, such as delusions, hallucinations, agitation, and catatonic behavior, and negative psychotic symptoms including blunted affect, anhedonia, avolition, and inattention (6, 33). Evidence from MRS and microdialysis studies has shown that PCP or ketamine and other antagonists of NMDAR can increase glutamate release (34–37). To verify that glutamatergic system dysfunction is involved in the psychopathology of schizophrenia, a large number of studies on glutamate levels in the brain were conducted through MRS, and according to the ketamine or PCP model, glutamate concentrations in the brain should increase in first-episode schizophrenia patients. Supporting the ketamine model, recent studies based on samples of first-episode schizophrenia have observed glutamate concentrations in the frontal, prefrontal, and anterior cingulate cortexes (38), and the striatum (26, 39). Contrary to this hypothesis, some studies found reductions in glutamate concentrations in the ACC (16) and the medial frontal cortex of first-episode schizophrenia patients (40). In our study, there were no significant differences in glutamate levels in the ACC between drug-naïve first-episode schizophrenia patients and healthy controls. The inconsistency in these studies may suggest that the ketamine model does not accurately reflect the mechanisms underlying schizophrenia.

Accumulating evidence has shown that glutamatergic dysfunction may be involved in the pathogenesis of schizophrenia, and glutamate levels may be associated with the outcome of schizophrenia and the severity of psychiatric symptoms (41, 42). In our study, we found lower Glu/Cr+PCr was correlated with more severe negative symptoms as assessed by the PANSS at baseline. Some studies with samples of chronic schizophrenia patients report that the severity of negative symptoms was associated with lower levels of Glu levels in the brain (41, 43, 44). However, in contrast to our results, several studies found that more severe negative symptoms were associated with higher levels of Glu in first-episode psychosis (16, 45, 46). The inconsistencies were most likely due to the effects of age, antipsychotic administration, and disease stage on glutamate concentration. Natsubori et al. (47) found that the Glx level of chronic and medicated schizophrenia patients in the mPFC and ACC was lower than that in the healthy control group, patients at ultrahigh risk for psychosis, and patients with first-episode schizophrenia. One study found that Glu levels in the ACC were significantly lower than those at baseline in patients with first-episode schizophrenia after 4 weeks of amisulpride administration (46). Therefore, demographic characteristics of different samples can influence the Glu level, which affects the determination of the correlation between Glu levels and symptoms. In the present study, our subjects were younger than those in many studies and had never taken antipsychotic drugs. Recently, a study (48) with samples that had a similar mean age with ours (22.3 ± 4.4 years) found that the Glu level positively correlated with the cognitive function of the patients. According to our results, a lower level of Glu/Cr+PCr indicates more severe negative symptoms in patients with schizophrenia in the early stage before treatment. ACC glutamate levels were associated only with negative symptoms, suggesting that lower glutamate levels are linked to poor clinical outcomes.

Longitudinal studies on the relationship between antipsychotic response and Glu levels before treatment are rare. Recently, a multi-center ^1^H-MRS study found that first-episode schizophrenia patients with a worse response to amisulpride had higher ACC glutamate levels at baseline before treatment (46). The advantage of this study was that amisulpride has high selectivity to dopamine D2 and D3 receptors (49, 50); thus, in schizophrenia patients, Glu levels could be linked to the dopamine-blocking effect, rather than to the effect of blocking other receptors. However, this study did not require that the patients taking part in the study had never taken the antipsychotics, and they were also examined without a withdrawal period before the experiment. Additionally, a large proportion of the patients in this study were users of other substances. These factors make it impossible to eliminate the possible effect of pre-study drug therapy and substance use on the patients' Glu levels and the outcome of amisulpride administration (51). In our study, we selected individuals with schizophrenia who had never been treated by antipsychotics and excluded substance users. We found that patients with a worse response to risperidone had lower Glu levels than patients with a better response. Binary logistic regression models showed that the overall discrimination accuracy of remission by ACC Glu and Glu/Cr+PCr was 77.1 and 74.3%, respectively. When the age and PANSS-total scores were introduced into the regression models, the discrimination accuracy of remission reached 85.7 and 82.9%, respectively, which also showed clinical significance.

Ketamine has been shown to induce schizophrenia-like symptoms (6, 33, 52). The advantage of the ketamine model is that the relative proportions of positive and negative symptoms induced by the drug are more similar to schizophrenia than the respective proportions of symptoms induced by amphetamine or LSD (33, 53). According to the hypothesis of the ketamine model, schizophrenia patients should have higher levels of glutamate, which is associated with more severe psychiatric symptoms. However, this popular model is not without its limitations. First, some studies have found that ketamine also has a high affinity for D2 receptors (54, 55), while a recent study found that ketamine has no affinity for D2 receptors (56). Other studies have found that ketamine can change the level of glutamate in the brain of humans or animals (57), so the psychotomimetic effects of ketamine may be related to the dopaminergic system. Second, schizophrenia is considered a neurodevelopmental disorder in which the nervous system undergoes a long process to reach the disease state (58). Therefore, it is not reasonable to study the pathological process of schizophrenia using the more acute ketamine model. Third, many results based on MRS, including ours, are inconsistent with or even wholly contrary to the hypothesis of the ketamine model (16, 44, 48). Therefore, future studies on glutamate levels based on MRS should not only rely on the hypothesis of the ketamine model but should also be considered from multiple perspectives.

Our study also had some limitations. First, we did not perform ^1^H-MRS scans on patients at week 8, so we could not evaluate the changes in glutamate concentration caused by risperidone, which was not considered in our initial design. Second, we assessed only the psychiatric symptoms by the PANSS but did not evaluate the cognitive and social functions, so we could not fully judge the overall functional status and outcome of the drug-naïve FES patients. Third, because ^1^H-MRS scans require individuals to be relatively calm, we excluded patients with underlying impulsivity, agitation, and excitement symptoms, so our sample was not representative of all first-episode schizophrenia patients. Fourth, our sample size is small, and the lower limit of the age of the inclusion criteria was relaxed to 16 years old, so the influence of age on Glu concentration cannot be excluded entirely. In addition, non-remission patients were younger, had more severe symptoms, and were consequently treated with higher doses of risperidone. The difference in risperidone dosage will affect the final outcome of the patient. Fifth, we asked only the patient himself/herself and his/her guardians about the patient's use of tobacco, alcohol, and substances. We did not perform urine or blood tests on each subject to determine whether they used tobacco, alcohol, or other substances. Thus, we cannot guarantee the authenticity of the above reported data.

In conclusion, we observed that there were no significant differences in baseline Glu or Glu/Cr+PCr in the ACC between drug-naïve FES patients and HCs. However, we found that lower Glu/Cr+PCr levels were highly associated with serve negative symptoms and that the patients with worse outcomes had lower baseline Glu levels. Our results suggested that baseline glutamate levels in the ACC may be used as a marker to evaluate the treatment effect of antipsychotics in schizophrenia patients.

## Materials and Methods

### Participants

We recruited 39 drug-naïve FES subjects from outpatients in the Second Xiangya Hospital, Central South University, China, and 42 HC subjects through advertisements posted at the same time. All FES patients were first diagnosed with schizophrenia using the *DSM-IV* criteria with a Mini-International Neuropsychiatric Interview (59) by two experienced senior psychiatrists. The inclusion criteria of all subjects were as follows: (1) right-handed Han Chinese individuals aged 16–30 years; (2) education≧6 years; (3) no prior exposure to antipsychotics; (4) no history of substance use; and (5) no major medical or neurological illness. None of the HC subjects met the diagnostic criteria for any mental illness in the *DSM-IV* or had a history of mental illness or a family history of mental disorders. Data from four patients (two lost to follow-up and two with MR contraindications) and two healthy controls (one with MR contraindications and one with an anatomical abnormality on structural scan) were excluded. Data from 35 FES patients and 40 HCs were included in the final analyses. The study was approved by the Second Xiangya Hospital Ethics Committee (No. S008,2012). All participants were aware of the detailed procedures of our study and signed informed consent forms.

### ^1^H-MRS

**Figure 3:**
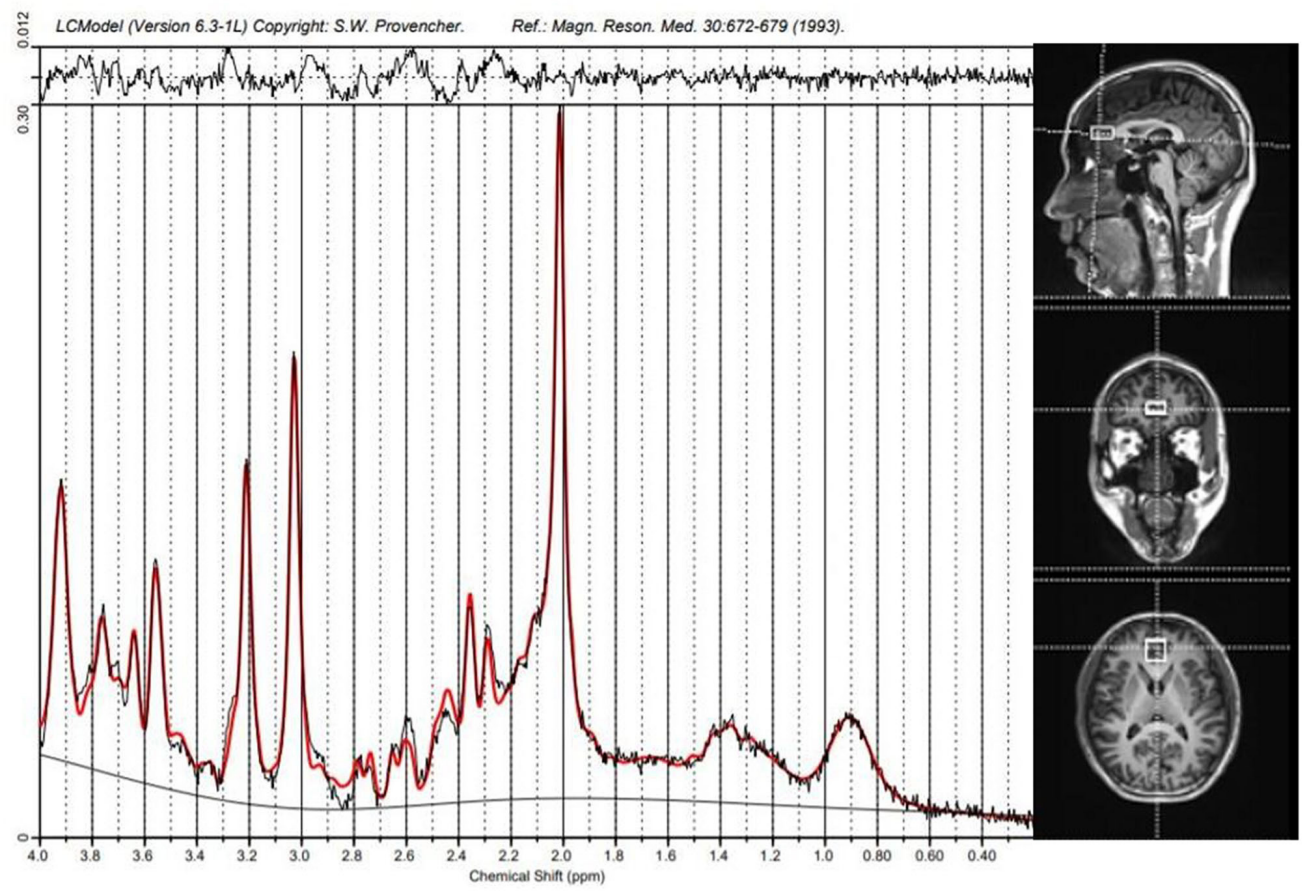
Spectroscopic voxel placement and proton magnetic resonance (^1^H-MRS) spectra. Voxel placement in the anterior cingulate cortex (ACC) and Sample ^1^H-MRS spectra acquired from ACC by LCModel.

All subjects were scanned in a 3T scanner (Siemens, Verio, Germany) with a 16-channel head coil at the Magnetic Imaging Centre of Hunan Children's Hospital, China. Scans of patients were performed before treatment with antipsychotics, while healthy controls had no time limit on undergoing the scan. Patients with acute psychotic symptoms underwent an ^1^H-MRS scan within a few hours and took risperidone immediately after the scan was completed. Patients with predominantly negative symptoms had an ^1^H-MRS scan later in the day, taking risperidone in the evening or morning the next day. Participants wore specially made foam pads to reduce head motion and scanning noise. T1-weighted anatomical MRI images were acquired with 3-dimensional magnetization-prepared fast gradient echo sequences (TR/TE = 2530 ms/2.33 ms; FOV = 256 × 256 mm; slice thickness = 1.0 mm; gap = 0 mm; NEX = 1; and 192 sagittal slices). Target voxels were placed in the ACC (10 × 20 × 20 mm), and the ^1^H-MRS data were acquired using a point-resolved spectroscopy sequence (PRESS) (svs_se; TR = 3000 ms; TE = 30 ms; NEX 80) (Figure 3).

^1^H-MRS spectra were analyzed with a linear combination model (LCModel version 6.3–1B) (Figure 3) at the Second Affiliated Hospital, Shantou, China. The cerebrospinal fluid in the target voxels was used as the internal reference to calculate the absolute concentration of Glu and Cr+PCr. The FWHM and SNR were checked to guarantee the quality of the MRS data, and only those spectra with an FWHM <0.1 ppm and an SNR> 10 were retained. Furthermore, data with a Cramer–Rao minimum variance ≥ 20% were discarded.

### Medication

All patients were taking antipsychotics for the first time and were treated with risperidone monotherapy for eight weeks following the scanning. It is contraindicated for co-use with mood stabilizers and antidepressants. None of the patients dropped out of the study because of severe side effects.

### Clinical Assessments

We assessed the severity of illness at both baseline and week 8 using the 30-item Positive and Negative Syndrome Scale (PANSS) (60). The assessment was carried out by two psychiatrists (interrater reliability score > 0.8). Remission status was defined by the criteria proposed by the Remission in Schizophrenia Working Group (29), but it did not include the six-month observation period. This standard requires that the score of the following items of the PANSS be less than 3: PANSS-Positive (P1, P2, P3), PANSS-Negative (N1, N4), PANSS-General (G5, G9).

### Statistical Analysis

We performed all statistical analyses in SPSS (version 20 IBM Inc., New York, USA), with statistical significance indicated by two-tailed *p*-values < 0.05. Group differences in demographical characteristics, levels of Glu and Glu/Cr+PCr, and PANSS scores were assessed using independent-samples *t*-tests or chi-square tests. Relationships between metabolite levels and baseline symptom scores of the PANSS were described with Pearson's correlation analysis. The association between baseline metabolite levels of Glu and Glu/Cr+PCr and patient outcome (remission and non-remission) at week 8 was analyzed with binary logistic regression.

## Data Availability Statement

The raw data supporting the conclusions of this article will be made available by the authors, without undue reservation.

## Ethics Statement

The studies involving human participants were reviewed and approved by The Second Xiangya Hospital Ethics Committee. Written informed consent to participate in this study was provided by the participants' legal guardian/next of kin.

## Author Contributions

JL, JT, HH, and XC designed the study. JL, ZL, HR, DW, XM, and LY collected the samples and clinical information. CL carried out the brain scanning. JL, HR, ZL, HH, JZ, and JT analyzed and discussed the experimental result. JL and HR wrote the first draft of the manuscript. All authors contributed to and have approved the final manuscript.

## Conflict of Interest

The authors declare that the research was conducted in the absence of any commercial or financial relationships that could be construed as a potential conflict of interest.

## Abbreviations

^1^H–MRS: proton magnetic resonance spectroscopy
ACC: anterior cingulate cortex
Glu: glutamate
Cr: creatine
PCr: phosphocreatine
CRLBs: Cramér Rao lower Bounds
FWHM: full width at half maximum
SNR: signal-noise ratio
HC: healthy control
FES: first-episode schizophrenia
PANSS: Positive and Negative Syndrome Scale
NMDAR: N-methyl-D-aspartate receptor
RM: remission
NRM: non-remission.
