# Supplementary material for: Anterior Cingulate Cortex Glutamate Levels Are Related to Response to Initial Antipsychotic Treatment in Drug-Naive First-Episode Schizophrenia Patients
Source: Front Psychiatry. 2020 Oct 23;11:553269. doi: 10.3389/fpsyt.2020.553269 (PMC7644538; doi:10.3389/fpsyt.2020.553269)
Supplement: Supplementary file 1 [file Table_1.DOCX]

**Supplement Table 1 Comparison between sexes**

|  | Control | Patients | P-value |
| --- | --- | --- | --- |
| Glu of males,  mean±SD | 8.672± 0.983(N=25) | 8.989±1.196(N=22) | 0.325 |
| Glu/Cr+PCr of males, mean±SD | 1.574±0.175(N=25) | 1.633±0.257(N=22) | 0.353 |
| Glu of females, mean±SD | 8.978±0.769(N=15) | 8.808±1.089(N=13) | 0.634 |
| Glu/Cr+PCr of females, mean±SD | 1.671±0.168(N=15) | 1.688± 0.306(N=13) | 0.860 |

**Abbreviations:** Glu, Glutamate; Cr, creatine; PCr, phosphocreatine
